# Balance of Interactions Determines Optimal Survival in Multi-Species Communities

**DOI:** 10.1371/journal.pone.0145278

**Published:** 2015-12-28

**Authors:** Anshul Choudhary, Sudeshna Sinha

**Affiliations:** Department of Physical Sciences, Indian Institute of Science Education and Research (IISER) Mohali, Knowledge City, SAS Nagar, Sector 81, Manauli PO 140 306, Punjab, India; National Scientific and Technical Research Council (CONICET)., ARGENTINA

## Abstract

We consider a multi-species community modelled as a complex network of populations, where the links are given by a random asymmetric connectivity matrix *J*, with fraction 1 − *C* of zero entries, where *C* reflects the over-all connectivity of the system. The non-zero elements of *J* are drawn from a Gaussian distribution with mean *μ* and standard deviation *σ*. The signs of the elements *J*_*ij*_ reflect the nature of density-dependent interactions, such as predatory-prey, mutualism or competition, and their magnitudes reflect the strength of the interaction. In this study we try to uncover the broad features of the inter-species interactions that determine the global robustness of this network, as indicated by the average number of active nodes (i.e. non-extinct species) in the network, and the total population, reflecting the biomass yield. We find that the network transitions from a completely extinct system to one where all nodes are active, as the mean interaction strength goes from negative to positive, with the transition getting sharper for increasing *C* and decreasing *σ*. We also find that the total population, displays distinct non-monotonic scaling behaviour with respect to the product *μC*, implying that survival is dependent not merely on the number of links, but rather on the combination of the sparseness of the connectivity matrix and the net interaction strength. Interestingly, in an intermediate window of positive *μC*, the total population is maximal, indicating that too little or too much positive interactions is detrimental to survival. Rather, the total population levels are optimal when the network has intermediate net positive connection strengths. At the local level we observe marked qualitative changes in dynamical patterns, ranging from anti-phase clusters of period 2 cycles and chaotic bands, to fixed points, under the variation of mean *μ* of the interaction strengths. We also study the correlation between synchronization and survival, and find that synchronization does not necessarily lead to extinction. Lastly, we propose an effective low dimensional map to capture the behavior of the entire network, and this provides a broad understanding of the interplay of the local dynamical patterns and the global robustness trends in the network.

## Introduction

Complex networks provide a common framework to address a vast range of phenomena in large interactive systems [1]. The use of network theory in studying the stability and dynamics of model ecosystems started with the landmark paper of Robert May [2] and the success and effectiveness of such inquiry can be gauged from the fact that even today most studies in theoretical ecology heavily rely on the network framework [3]. Our current understanding of the stability of an ecological network hinges around two key aspects: *interaction topology* and *nature of interactions*. Now, there are wide ranging situations where one either does not have sufficient information on the exact underlying topology, or one finds that the web of interactions essentially appears to be a random network. In such cases, the interactions are modelled by random connectivity matrices, and the broad nature of interactions is our only guiding principle in analyzing the dynamics and survival properties of the complex system.

In this study, we are going to explore the effect of the balance of different kinds of interactions in a multi-species community on the collective dynamical behaviour of the network [4]. Our focus will be on the *global robustness* of the system, as exemplified by the total population of all species [5]. It is evident that the total population reflects the global state of the network effectively, while being sensitive to the underlying dynamics at the local species level as well. So here we will explore how *diversity in interactions influence the emergent dynamics*, and the *relation of these dynamical patterns to survival of populations*, lending yet another perspective to the stability-diversity debate.

Recently, Mougi & Kondoh [6] studied the interesting effects of diversity in interaction types on the stability of an ecological community and they found that diversity is a key element in determining stability and biodiversity. However their results are based on linear stability analysis for small perturbations about a local equilibrium, and they do not give the relationship between survival and the emergent dynamical patterns. In this context, our study provides a complementary exploration of the global survival features of such systems [7] and also relates it to dynamical behavior of the constituent populations.

## Model

The model we consider here is inspired by the earlier theoretical studies conducted by Robert May [2, 8]. However, we would like to mention here that unlike most studies regarding stability [6, 9] which assume the existence of time-independent population densities when system reaches steady state, *we consider the more general condition where the attracting state can have complex temporal behavior*, rather than a fixed point solution [7]. the principal motivation for our approach to the question of stability is its wider relevance and broader applicability. Further, rather than local stability about an equilibrium, we will focus on a different set of *global quantifiers of robustness and survival in the complex network*.

Specifically, in this work we consider a prototypical map, the Ricker (Exponential) Map, modelling population growth of species with non-overlapping generations:

$$f\left(x\right)=x\;e^{r(1-x)}$$ (1)

where *r* is interpreted as an intrinsic growth rate and (dimensionless) *x* is the population scaled by the carrying capacity.

We then consider the evolution of *N* such interacting populations given as:

$$x_{i}\left(n+1\right)=f\left(x_{i}\left(n\right)\right)+\frac{1}{N}\sum_{j}J_{ij}x_{j}\left(n\right)$$ (2)

where *i* = 1, … *N*, and the connectivity or community matrix, *J* represents how species are mutually interacting. Further we consider that *x*_*i*_(*n* + 1) = 0 if *x*_*i*_(*n* + 1) < *x*_*threshold*_, where *x*_*threshold*_ is the minimum population density below which population cannot sustain on their own and therefore will eventually become extinct, namely the Allee Effect [10].

The connectivity matrix *J* is a random asymmetric matrix, with fraction 1 − *C* of zero entries, where *C* reflects the over-all connectivity of the system. The non-zero elements are drawn from a Gaussian distribution, $\frac{1}{\sqrt{2\pi \sigma^{2}}}e^{\frac{-{\left(x-\mu \right)}^{2}}{2\sigma^{2}}}$, with mean *μ* and standard deviation *σ*.

The signs of the elements *J*_*ij*_ of the connectivity matrix *J* reflects the nature of density-dependent interactions. In general, neutral interactions are reflected by zero matrix elements. Interactions that reduce the population at a node, for instance through parasitism, grazing, and predation, will be reflected by a negative sign, while interactions that benefit a species, for instance by providing refuge from physical stress, predation or competition, will bear a positive sign.

So then, when we have mutualism or symbiosm, both *J*_*ij*_ and *J*_*ji*_ are positive. When we have competition or antagonistic interactions, both *J*_*ij*_ and *J*_*ji*_ are negative. When the effect of one species on the other is positive, but neutral the other way round, we have commensalism, reflected by *J*_*ij*_ > 0 and *J*_*ji*_ = 0. Similarly, amensalism is reflected by *J*_*ij*_ < 0 and *J*_*ji*_ = 0. General predator-prey interactions are captured by *J*_*ij*_ and *J*_*ji*_ having different signs.

In general, positive interactions or facilitative interactions between species that benefit the growth of the species, give rise to more positive mean *μ*. Namely, high positive *μ* implies the dominance of mutualism in the ecosystem where as *μ* ≈ 0 would imply balance of different kinds of interactions in the system. The standard deviations, *σ* on the other hand controls the degree of variability in the strength of these interactions. Finally, connectedness, *C* is another important factor that tell us how many species are interacting with each other. Namely, it reflects the fraction of neutral interactions in the network.

So, to summarize, we consider a system (Eq 2) with the following premises:

1. We have a network of interacting well mixed populations.
2. The dynamics at the nodes of the network is chaotic, reflecting the intrinsic complexity of the population dynamics.
3. The population dynamics display the Allee effect and cannot sustain itself below a threshold level. So when a population is labeled extinct, it implies that the population has dropped below a threshold. So a population with value zero in Eq 2, represents a sub-threshold population that will eventually die out, unless there is enough cooperative help from other species in the network. Further, the sub-threshold population does not have any contribution in the interaction term, as its numerical value is zero. Therefore a sub-threshold population can be affected by other populations but has no impact on others until it becomes above-threshold again.
4. The single uncoupled population is prone to extinction (namely will become sub-threshold).
5. However in an interactive network of populations, a population falling to sub-threshold levels locally, may revive through cooperative effects of other species. So clearly in our model it is possible obtain persistent communities with above-threshold populations only through suitable interactions among the species. This is reminiscent of the “rescue effect” in models of metapopulations.

The main aim of this study is to *understand the relation between broad features of the interaction matrix and the collective dynamics of the system, and then go on to link this to the local and global survival in the system*.

## Methods

At the outset, we present our tools and describe the measures for analyzing the survival properties of the system. To gauge the robustness of the system, we first calculate the *number of active nodes*, namely the number of non-extinct species with non-zero population, after transience. This quantity is then averaged over a period of time *T* and further averaged over *N*_*ic*_ different initial conditions. We denote this averaged number of non-extinct nodes by 〈*N*_*active*_〉, and it is defined as:

$$\left\langle N_{active}\right\rangle =\frac{1}{N_{ic}T}\sum_{N_{ic}}\sum_{T}\;\left\{\sum_{i=1}^{N}\varphi_{i}\left(t\right)\right\}\;\;\;\text{where},$$ (3)

$$\varphi_{i}\left(t\right)=\left\{\begin{array}{cc} 1 & \;\;\;\;\;\text{if}\;x_{i}\left(t\right)>x_{threshold}\text{,}\\ 0 & \;\;\;\;\;\text{otherwise,} \end{array}\right.$$ (4)

The next important measure of global survival is the *total population* $\Sigma_{i=1}^{N}x_{i}$, of the system, reflecting the biomass yield in a multi-species community. This quantity, averaged over a period of time *T* and over *N*_*ic*_ different initial realizations, is denoted by 〈*x*_*total*_〉, and mathematically expressed as:

$$\left\langle x_{total}\right\rangle =\frac{1}{N_{ic}T}\sum_{N_{ic}}\sum_{T}\;\left\{\sum_{i=1}^{N}x_{i}\left(t\right)\right\}$$ (5)

### Synchronization Order Parameter

In order to probe collective patterns in the network, we studied the level of synchronization that emerges in the system. To quantify the degree of synchronization we have employed two different order parameters.

1. *Z*_*sync*_: Here we measure synchronization error as the mean square deviation of the local state of the nodes, averaged over time *T* (after transience) and over *N*_*ic*_ different realizations [11–13], mathematically expressed as:
  $$Z_{sync}=\frac{1}{N_{ic}T}\sum_{N_{ic}}\sum_{T}\;{\left\{\frac{1}{N}\sum_{i=1}^{N}\left.[x_{i}\left(t\right)-\left\langle x\left(t\right)\right\rangle \right]\right.}^{2}$$ (6)
  When this measure goes to zero, it reflects *complete synchronization* in the system.
2. *Z*_*phase*_: This is a phase order parameter that reflects the degree of variation in the phases of the local dynamics at the nodes. Specifically, it is a measure of the fraction of nodes in the largest phase cluster, averaged over time *T* and over different network realizations *N*_*ic*_. When *Z*_*phase*_ = 1, it implies that the entire system is phase synchronized (though not necessarily in complete synchronization).

In particular we consider the specific case, observed over a large parameter range, where the local dynamics is a 2-cycle, given by values *x*_1_ and *x*_2_, or a chaotic band where the states lie in two bands bounded by ($x_{min}^{1}$, $x_{max}^{1}$) and ($x_{min}^{2}$, $x_{max}^{2}$). Here *Z*_*phase*_ then is the supremum of the quantity

$$\frac{1}{N_{ic}T}\sum_{N_{ic}}\sum_{T}\;\left\{\frac{1}{N}\sum_{i=1}^{N}\varphi_{i}\left(t\right)\right\}$$

where *φ*_*i*_(*t*) is 1 if *x*_*i*_(*t*) lies in the specified band (namely equal to *x*_1_/*x*_2_ or lying in the range [$x_{min}^{1}$:$x_{max}^{1}$]/[$x_{min}^{2}$:$x_{max}^{2}$]), and 0 otherwise.

So these measures provide complementary information about the synchrony in phase and amplitude of the dynamics of the local constituents of the network.

### System Parameters

In this work parameter *r* = 4 in Eq 1, namely the local map is in the chaotic regime, and the threshold value *x*_*threshold*_ = 0.0001. All results reported here are robust with respect to small variations around these values. The survival and synchronization measures were calculated by averaging over 100 random initial conditions, i.e. *N*_*ic*_ = 100 in the equations above. The system sizes ranged from 100 ≤ *N* ≤ 800, with connectedness 0 ≤ *C* ≤ 1 and standard deviation 0.1 ≤ *σ* ≤ 0.5 in the connectivity matrix *J*. Further, to explore the effect of the mean interaction strength *μ* on the dynamics, which is a focus of our work here, we investigated the range: −1 ≤ *μ* ≤ 1. Note that several earlier studies have been confined only to the balanced situation, i.e. *μ* = 0.

In the sections below, we present the principal observations from our extensive simulations over this wide-ranging window of parameters.

## Results and Analysis

### Survival in the Network

**Fig 1:**
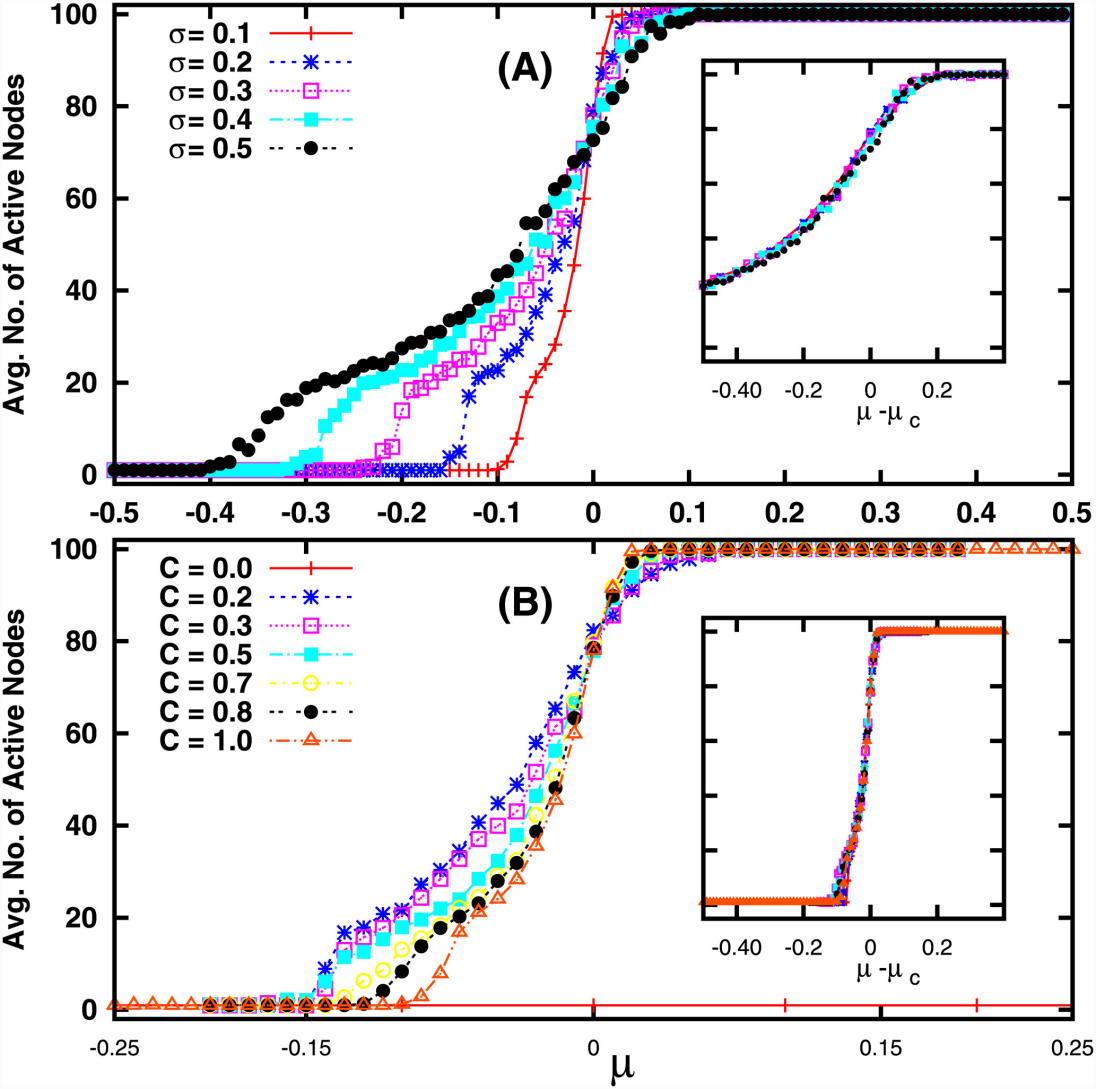
Average number of active nodes, 〈*N*_*active*_〉, as a function of mean *μ* of the interaction strengths of the connectivity matrix *J*, for different values of connectivity *C* and different variabilities in interaction strengths *σ*. Here system size *N* = 100, and so 〈*N*_*active*_〉 = *N* = 100 in the figures reflect a network where no species becomes extinct. The insets show the collapse of the scaled 〈*N*_*active*_〉, with respect to *μ* − *μ*_*c*_, where critical *μ*_*c*_ = 0.

We first calculate the average number of active nodes (namely the average number of non-extinct species) 〈*N*_*active*_〉, as a function of the mean interaction strength *μ* of the connectivity matrix *J*. As evident from the results displayed in Fig 1, the average number of active nodes 〈*N*_*active*_〉 in the network rises sharply as a function of mean interaction strength *μ* around *μ* ≈ 0. When the mean interaction strength is quite negative, the number of active nodes goes to zero, i.e. the entire system is driven to extinction. For positive *μ* all nodes in the network are non-zero, i.e. no species goes extinct. The connectedness *C* and the variability of interaction strengths *σ* then does not affect the number of active nodes in the network when the network is far from balanced, namely considerably positive mean interaction strengths yield 〈*N*_*active*_〉 = *N*, while considerably negative interactions results in 〈*N*_*active*_〉 = 0, irrespective of *C* and *σ*. The transition from complete extinction to a completely active network is sharper for systems with low variability in interaction strengths (i.e. low *σ*), and for systems with higher connectedness (i.e. high *C*).

To gain further quantitative understanding of the nature of this transition, we explored the scaling behaviour near the transition, and discovered that the average number of active sites scales with respect to *C* as:

$$\left\langle N_{active}\right\rangle \;∼\;\Theta \left(\left(\mu -\mu_{c}\right)C^{\alpha }\right)$$ (7)

Further the number of active sites scales with respect to *σ* as:

$$\left\langle N_{active}\right\rangle \;∼\;\Omega \left(\left(\mu -\mu_{c}\right)\sigma^{\beta }\right)$$ (8)

Here, *α* and *β* are appropriate critical exponents for scaling functions Θ and *Ω* respectively. A good data collapse, shown in Fig 1 (insets), is obtained for *μ*_*c*_ = 0, with *α* = 0.45 ± 0.02 and *β* = 1. These scaling relations suggest that a transition from complete extinction to a fully active ecosystem occurs around *μ* = 0, namely around the state of balanced interaction strengths or completely neutral interactions [14]. We also performed finite size scaling with respect to system size *N*, and found the simple scaling: 〈*N*_*active*_〉∼*Nf*(*σ*, *μ*, *C*), implying that the active fraction 〈*N*_*active*_〉/*N* is independent of *N*.

**Fig 2:**
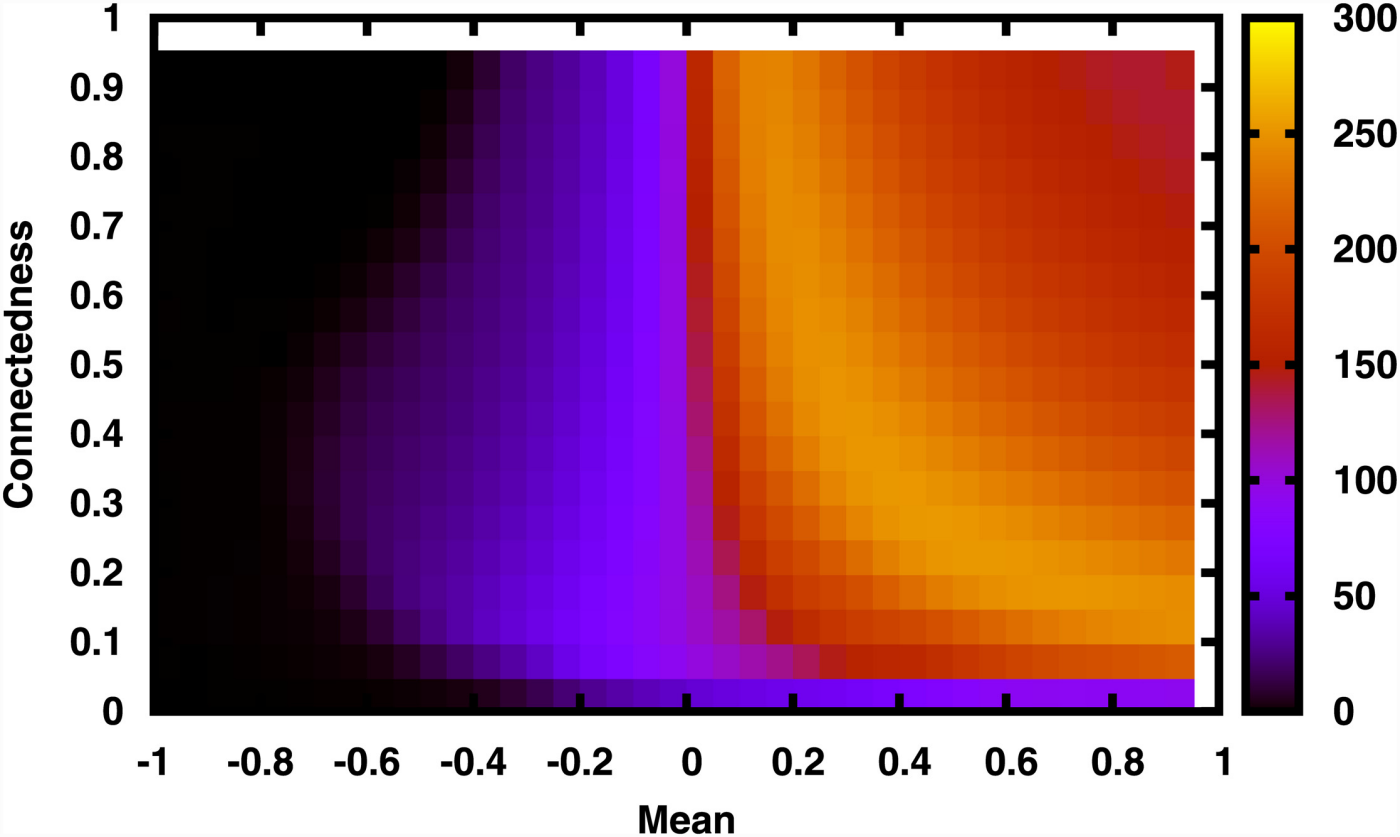
Average total population 〈*x*_*total*_〉 of a system of size *N* = 100 (represented by the color scale), as a function of mean *μ* of the interaction strengths and the connectedness *C* (giving the number of non-zero entries in connectivity matrix *J*). Here standard deviation *σ* of the non-zero matrix elements is 0.5.

**Fig 3:**
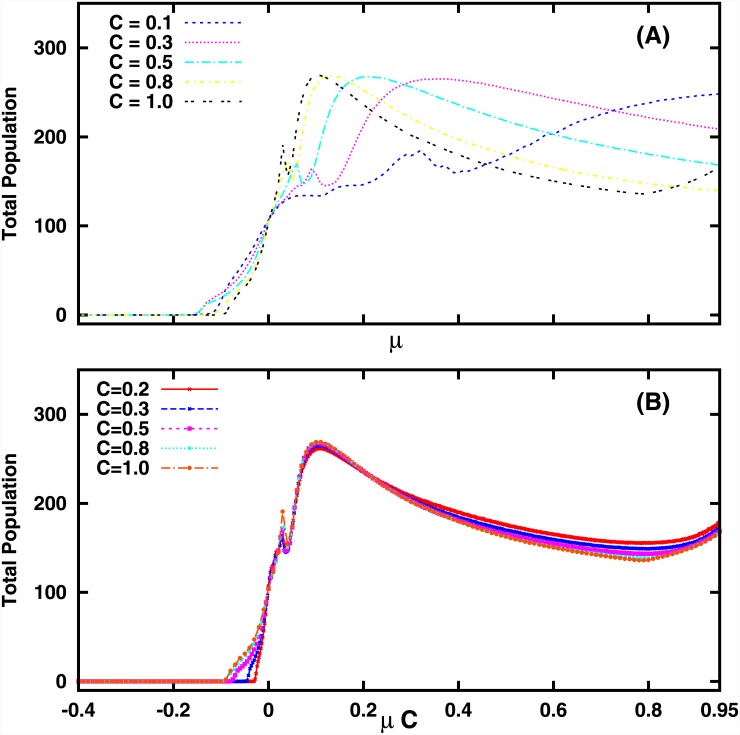
Average total population of 〈*x*_*total*_〉 of a system of size *N* = 100, as a function of mean *μ* of the interaction strengths for (A) different values of connectedness parameter *C*. In (B), the average total population is given as a function of the product *μC*, and it is evident that the data collapses onto a single curve implying a functional relation between total population and *μC*. Here the average total population is obtained by averaging the total over 100 random realizations, and the standard deviation *σ* of the connectivity matrix *J* is 0.1.

The next important measure of global survival is the average total population 〈*x*_*total*_〉 of the system, reflecting the biomass yield in a multi-species community. The variation of 〈*x*_*total*_〉, as a function of mean *μ* of the interaction strengths and connectedness *C* of the interaction matrix *J*, is shown in Figs 2–3. It is clear that for *C* = 0, i.e, when there are no interactions, the local extinctions accumulate, eventually leading to mass extinction.

When interactions are present, different global scenarios emerge with respect to varying mean *μ* and connectedness *C* of matrix *J*. For fixed *C*, the total population increases sharply with increasing *μ*, namely with increasing net positive interactions, around *μ* ≈ 0. So we find that for networks close to the balanced situation, we have enhanced population densities indicating greater survival, for increasing net positive interactions.

Further, there exists an interval of mean *μ*, around *μ* ≈ 0, where the average total population always increases with the increase in the number of interactions among species, namely increasing *C*. This would imply that connectivity always enhances survival of the system here. However, when mean *μ* is smaller or larger than this interval, one finds that at *intermediate values of connectedness the total population* is the largest (cf. Fig 2). Namely, a mix of neutral interactions along-side other interactions is most conducive to enhanced total population yield.

There is a critical negative mean, $\mu_{c}^{extinction}$ (where $\mu_{c}^{extinction}$ is a function of *C*) for which the local species experience severe loss of population leading to global extinction. There is also a critical positive mean, $\mu_{c}^{explosion}$, where $\mu_{c}^{explosion}C\approx 1$, such that for mean $\mu >\mu_{c}^{explosion}$ the nodes experience unbounded and explosive growth, destabilizing the whole network. We consider $\mu <\mu_{c}^{explosion}$ in our study.

Interestingly, we uncovered a scaling pattern between the total population and characteristics of the connectivity matrix. The data collapse of the population onto a single *non-monotonic* curve in Fig 3b reveals a scaling relation between total population and the product of the mean interaction strength, *μ* and connectedness, *C* of the network. This implies that the most relevant quantity in understanding the global behaviour of the network is *μC*, rather than *μ* and *C* alone. So clearly, survival is dependent not merely on the number of links, but on the combination of the sparseness of the connectivity matrix and the net interaction strength. For instance, fewer interactions (i.e. low *C*) tends to decrease the population, but this effect may be compensated by more positive interactions, i.e. higher *μ*. More importantly, the existence of an intermediate window of positive *μC* where the total population is maximum indicates that too little or too much positive interactions is detrimental to survival. In fact survival is optimal when the network has intermediate net positive connection strengths. So counter-intuitively, if positive interactions such as mutualistic or symbiotic links dominate other kinds of interactions too much, its effect ceases to be beneficial, causing the total population to reduce.

### Local Dynamics

Now we attempt to correlate these global features to local species-level dynamics. Namely, we attempt to correlate the survival and global stability of the ecological network to dynamical patterns emerging in the network as a result of interactions.

**Fig 4:**
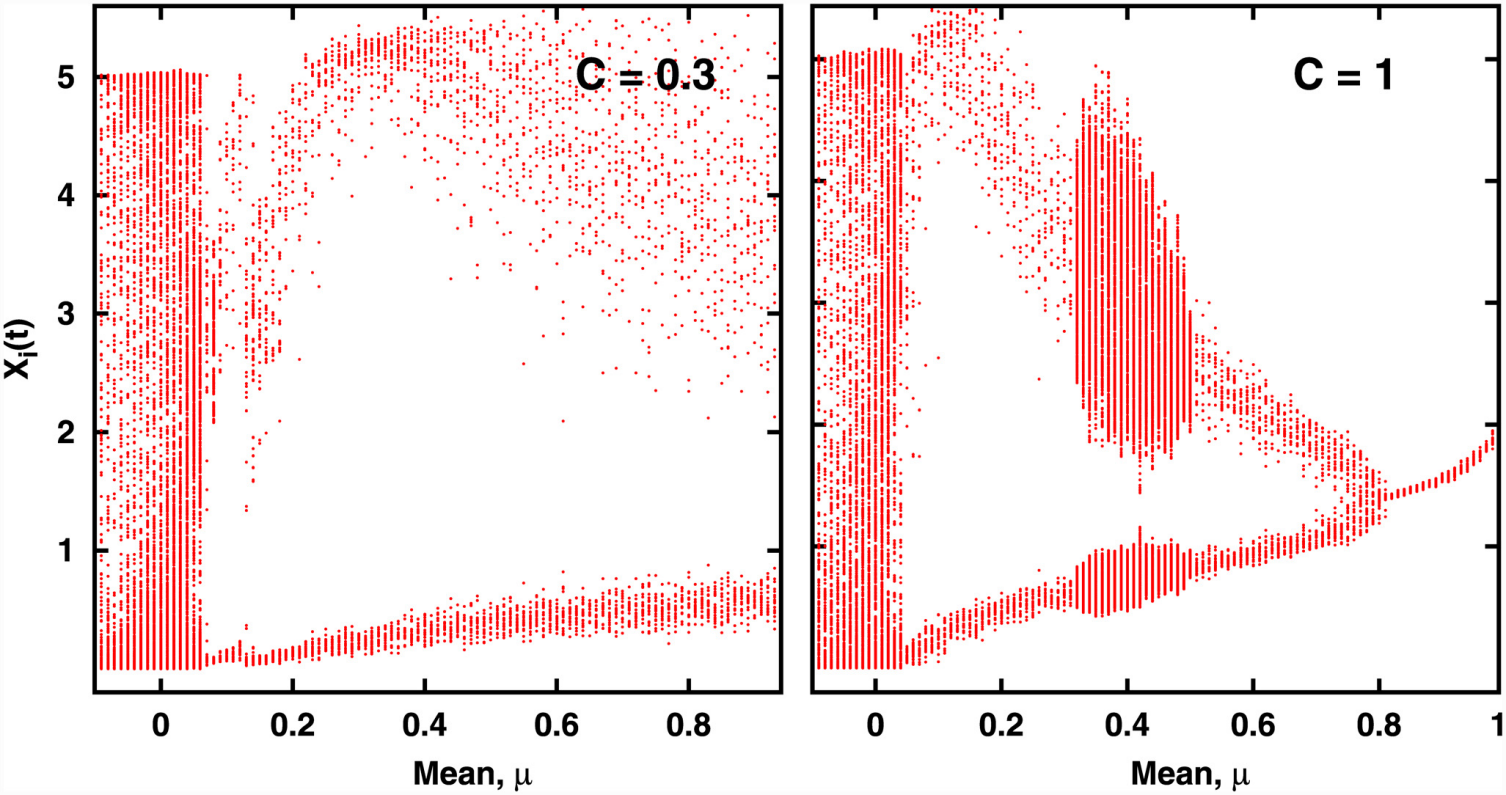
Bifurcation Diagram of the population dynamics of the network of 100 species, as a function of mean *μ* of the interaction strengths, for representative values of connectedness: (a) *C* = 0.3 and (b) *C* = 1. Here we show *x*_*i*_(*t*), for all *i* = 1, … *N*, over a period of time, after transience. Standard deviation *σ* of *J* is 0.1. On careful observation we found that all bifurcation diagrams collapse on to a single pattern when viewed as a function of *μC*.

From the bifurcation diagrams displayed in Fig 4, one can clearly discern the presence of coherent collective dynamics in the system. This coherence breaks down as one approaches *μ* = 0, as evident in Fig 4, with the network displaying unsynchronized chaotic behavior.

**Fig 5:**
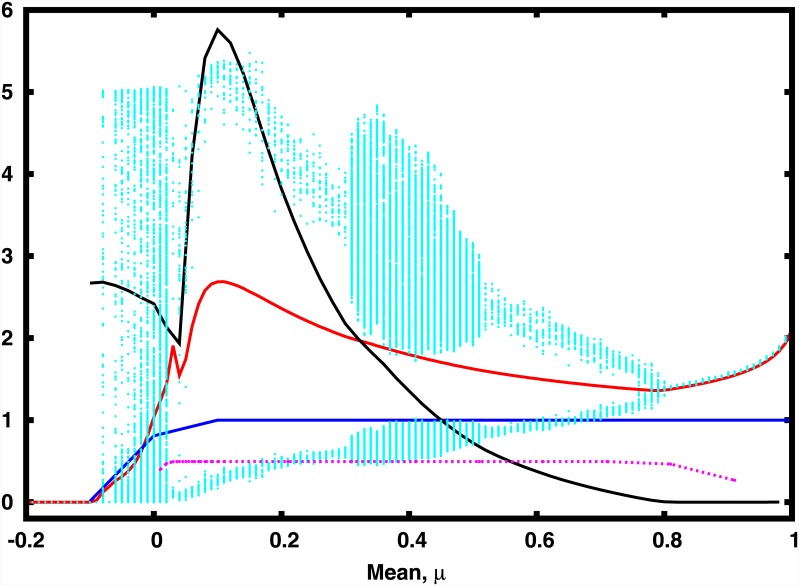
Synchronization error (dotted black), total population (red), bifurcation diagram for the emergent behavior of the population densities (cyan), the average number of active nodes (blue) and phase order parameter, *Z*_*phase*_ (magenta), as a function of mean *μ* of the interaction strengths. Here the connectivity matrix *J* has *C* = 1, *σ* = 0.1 and the size of network is *N* = 100. Note that the number of active nodes alone does not determine the total population, as a species can be active and yet persist at very low population densities.

In order to gauge the degree of synchronization among the nodes quantitatively we calculate the synchronization order parameter *Z*_*sync*_. Our attempt now will be to find the *correlation between synchronization and survival*. This is an important question, as synchronization has often been seen as increasing risks of extinction. Fig 5 exhibits this synchronization error, along side the number of active patches (i.e. nodes with non-zero population), the total population and collective dynamics of the whole network. It is clear that *synchronization does not necessarily lead to extinction*. In fact for positive mean interactive strengths, even when the entire system is completely synchronized, *all nodes are active*. The rationale for the above observation is that synchronization is a threat only when the synchronized dynamics covers a large range of population densities, such as in synchronized chaos, which typically is ergodic over state space. Here on the other hand, the synchronized dynamics is confined to the “safe zone” and the attractor trajectory does not enter the extinction region [15]. So the synchronized patches survive.

**Fig 6:**
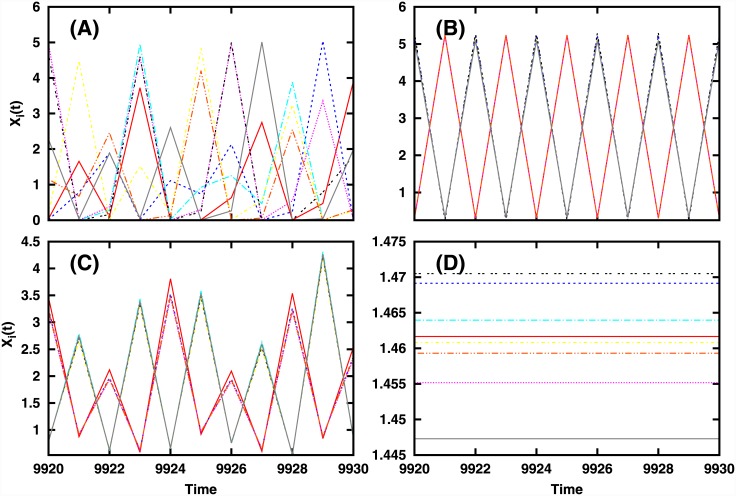
Time series of 10 representative sites in a network of 100 species, for different mean *μ* of the interaction strengths: (a) *μ* = 0 (b) *μ* = 0.12 (c) *μ* = 0.40 and (d) *μ* = 0.85. Here the connectivity matrix *J* has *C* = 1 and *σ* = 0.1.

We further investigate the nature of the time series of the local patches to discern cluster formation, and the phase relation between the clusters. We find that when the mean of the interaction strengths has a low positive value, the populations are attracted to a period 2 cycle, and the system divides into two anti-phase clusters (cf. Fig 6). Namely, alternately in time, one set of nodes in the network have low population densities, while the other set has high population densities. This behaviour is reminiscent of the field experiment conducted by Scheffer et al [16] which showed the existence of self-perpetuating stable states alternating between blue-green algae and green algae.

We also studied the phase clusters emerging in the system, by calculating a phase order parameter *Z*_*phase*_, which gives the fraction of species whose dynamics are in-phase in the network. This quantity is ≈ 0.5 in a large range of positive mean interaction strengths (*μ* ≈ 0.1 − 0.6), indicating that here the network always splits into two approximately equal clusters. In each cluster the nodes are in-phase with respect to each one another, and anti-phase with respect to nodes in the other cluster. However note that the degree of complete synchronization, which depends on both phase and amplitude, will be dependent on *μ* (as evident from Fig 5). So changing the mean interaction strength changes the nature of the dynamics without destroying this phase relationship and two phase synchronized clusters of varying amplitudes emerge in a large range of *μ*.

### Effective map for nodal dynamics

To gain further understanding of the dynamical patterns, we construct an effective map to mimic the essential dynamics of the nodes. Our approach is to split the interactive part in Eq 2 into an average part and a term capturing the fluctuations. Here the mean interaction strength, which is the dominant contribution, is *μC*, as there are a fraction *C* of non-zero interaction strengths drawn from a distribution with mean *μ*. So as first approximation, neglecting fluctuations, we can model the local dynamics as:

$$x_{eff}\left(n+1\right)=f\left(x_{eff}\left(n\right)\right)+\mu C\;x_{eff}\left(n\right)$$ (9)

when *x*_*eff*_(*n* + 1) > *x*_*threshold*_, and *x*_*eff*_(*n* + 1) = 0 otherwise.

Such an effective map is an accurate representation of the population dynamics when there is a high degree of coherence in the system. Namely, when there is complete synchronization in the network, all *x*_*i*_(*n*) ≡ *x*_*eff*_(*n*). Notice however that the effective map fails to capture the revival of sub-threshold populations due to positive cooperative effects of interactions, as *x*_*eff*_(*n*) < *x*_*threshold*_ leads to *x*_*eff*_ = 0 for all subsequent times.

**Fig 7:**
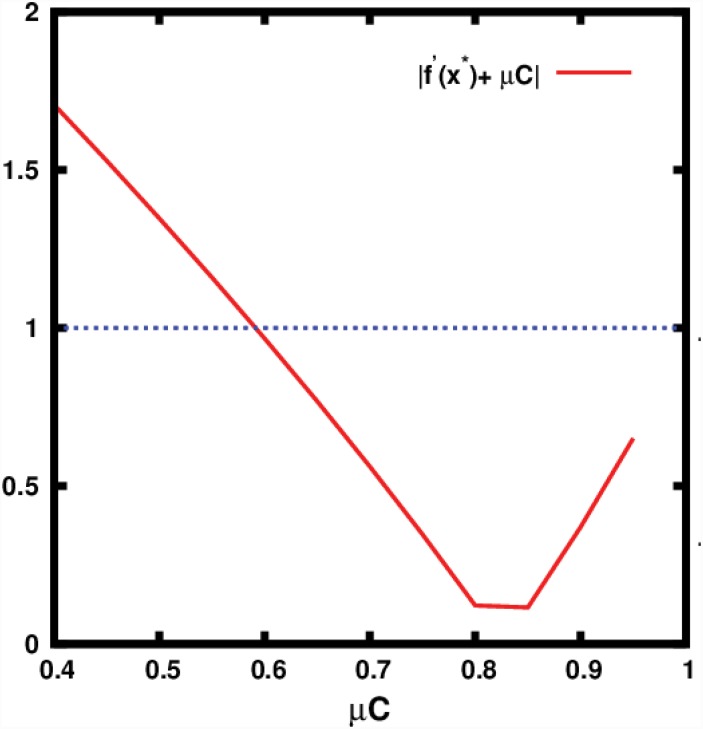
Stability curve for the fixed point obtained from Eq 10. The fixed point loses stability when |*f*′(x*) + *μC*|>1, and this occurs for *μC* ≈ 0.6, which is consistent with the results in Fig 8.

**Fig 8:**
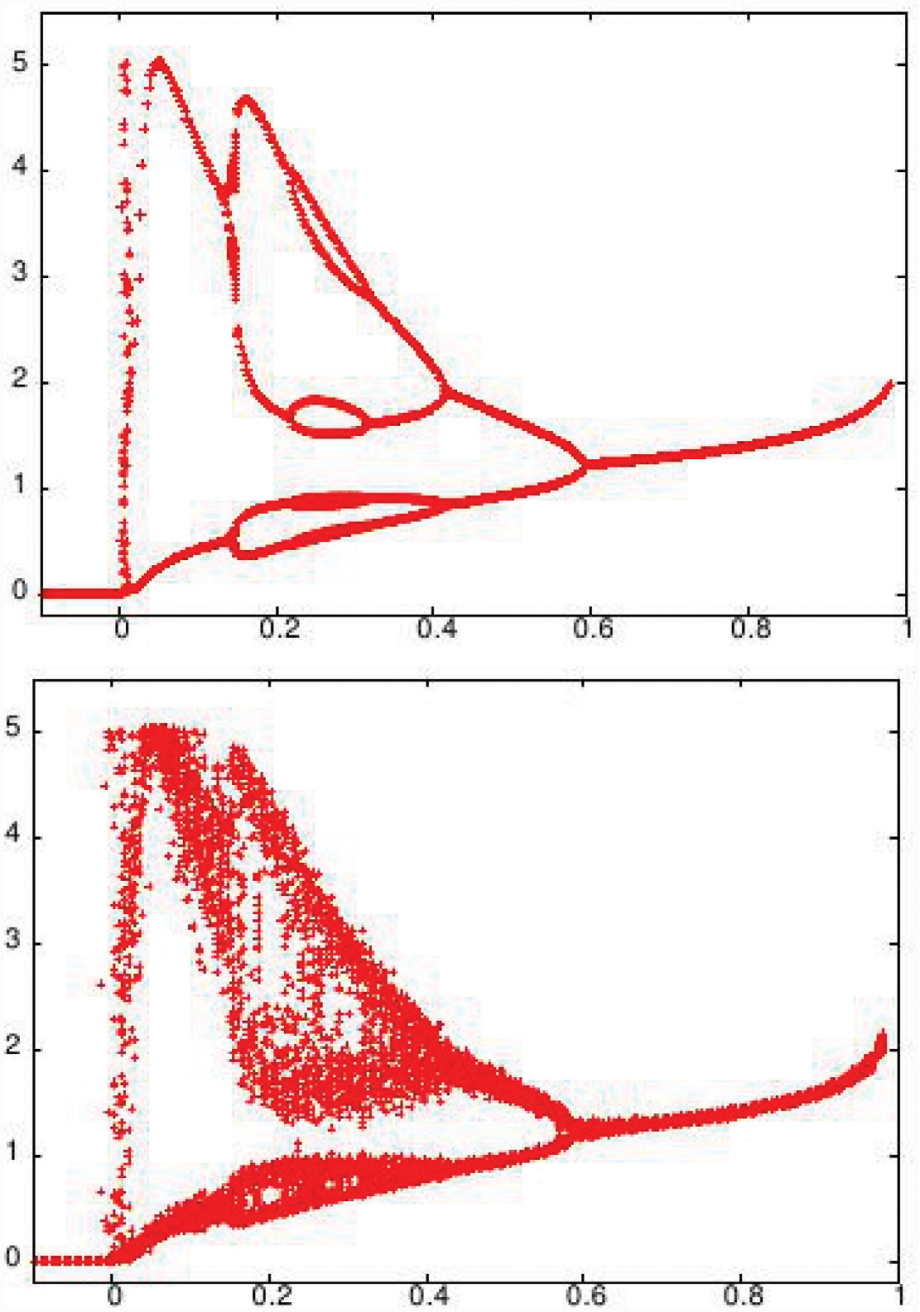
Bifurcation diagram of (top) effective map given by Eq 9, and (bottom) effective map under noise given by Eq 11, as a function of *μC*. In Eq 11 the random variable *η* is drawn from a zero-mean unit-variance Gaussian distribution, and *ξ* is drawn from a uniform distribution [−1 : 1] (as fluctuations in *x*_*i*_ are bounded). Specifically, *D*_*J*_ = 0.01 and *D*_*x*_ = 0.02.

*Analysis*: We can straight-forwardly analyze the effective map dynamics given by Eq 9 to find the windows where the positive steady state is stable. Note that the non-trivial fixed point, which is a function of *μC*, can be obtained as a solution of:

$$x_{eff}^{*}=\frac{1}{1-\mu C}f\left(x_{eff}^{*}\right)$$ (10)

and its stability is determined by the condition $|f^{\prime }\left(x_{eff}^{*}\right)+\mu C|<1$. Clearly as *μC* → 1, the fixed point becomes unboundedly large. This also explains the presence of the critical positive mean $\mu_{c}^{explosion}$, with $\mu_{c}^{explosion}C\approx 1$, in the system. From Fig 7 it is clear that the parameter yielding fixed populations in the system is very close to that found in the effective map (cf. Fig 8).

**Fig 9:**
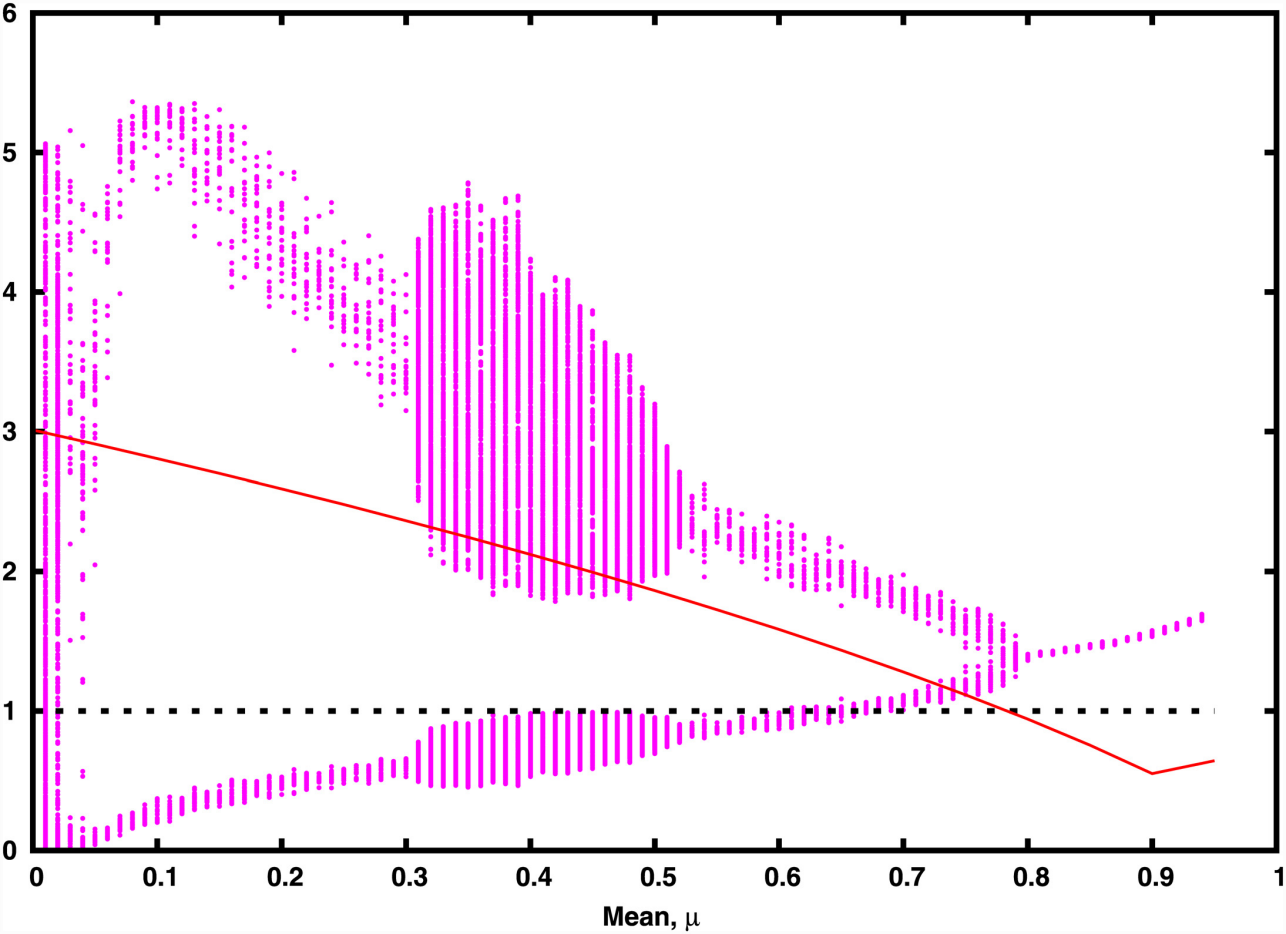
Bifurcation diagram of the population dynamics of all the species as a function of mean interaction strength *μ*, alongside the average of the extremal magnitude of the eigenvalues obtained by sampling a large number of random realizations of the connectivity matrix in Eq 2 (shown in red). The stability condition is satisfied when the eigenvalues are contained in the unit circle in the complex plane, namely when the red curve lies below 1 (indicated by the dotted black line).

Further, in order to obtain a better estimate of the stability of the synchronized steady state, we analyse the distribution of eigenvalues of the Jacobian corresponding to Eq 2 incorporating the full connectivity matrix. For the general case of asymmetric connectivity matrices, where the eigenvalues can be complex, the stability condition requires that the eigenvalues of the Jacobian matrix must lie within the unit circle in the complex plane. Fig 9 displays the bifurcation diagram along-side the extremal magnitude of the eigenvalues. From there it is evident that the parameter region where the steady state is stable, namely where the magnitude of the eigenvalues are bounded by 1, matches the value (*μ* ≈ 0.8) observed in simulations. Thus linear stability analysis provides an accurate range for the steady state of the network. However, we reiterate, the existence of the steady state is not directly related to global survival features, as evident through Fig 5.

Lastly we focus on the fixed point $x_{eff}^{*}=0$, namely an extinct state. Now in order to assess the size of the basin of attraction of $x_{eff}^{*}=0$, we determine the range of values of *x*_*eff*_ that can possibly map into the extinction zone. Namely, we try to ascertain the pre-images of sub-threshold *x*_*eff*_.

**Fig 10:**
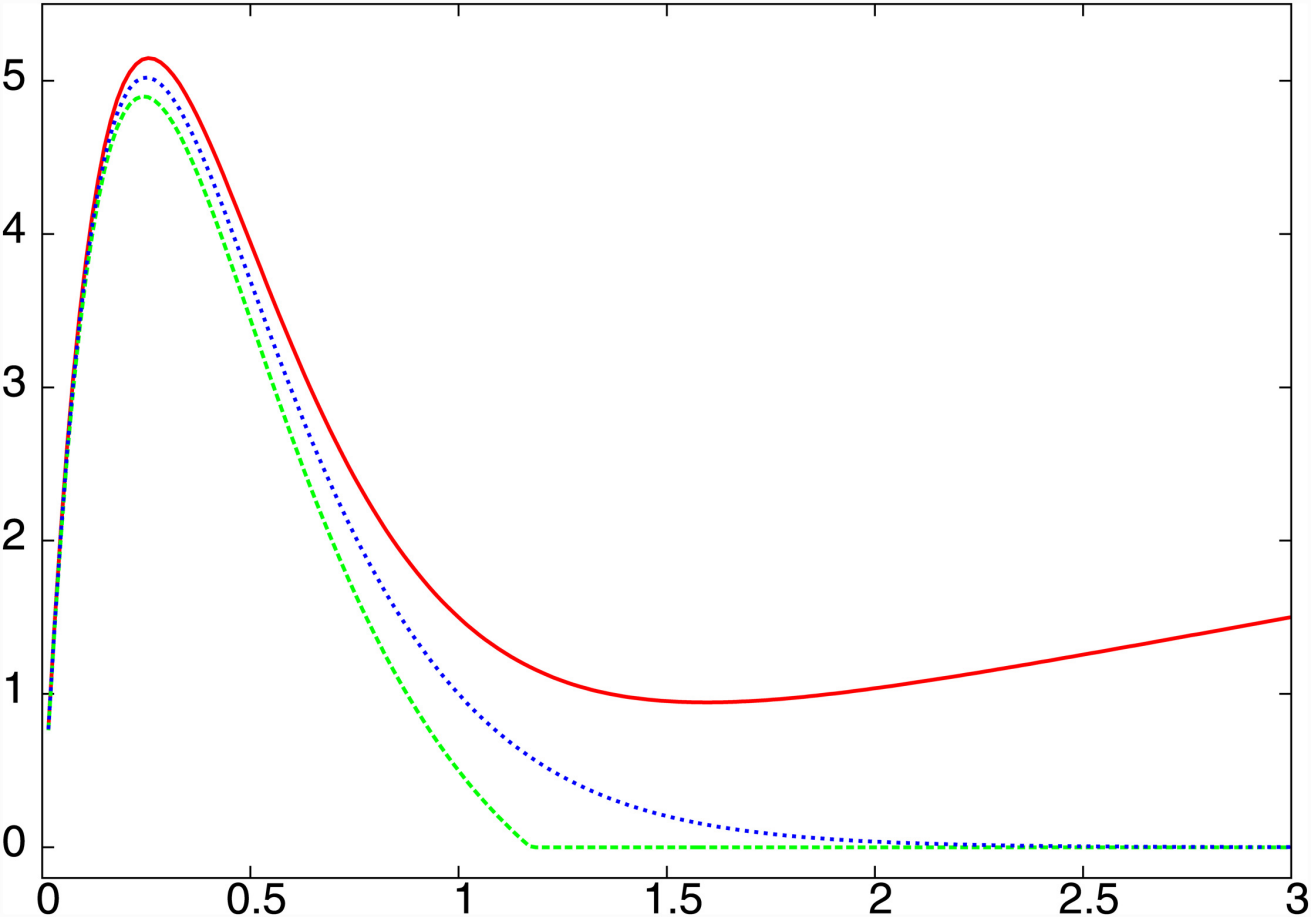
*x*_*eff*_(*n* + 1) vs *x*_*eff*_ given by the effective map in Eq 9, for *μC* equal to 0.5 (red), −0.5 (green) and 0 (blue). The threshold value *x*_*threshold*_ = 0.0001. It is clear that large *x*_*eff*_ never gets mapped to zero for positive *μC*, while for negative *μC* and for *μC* = 0 (uncoupled case) high *x*_*eff*_ maps to *x*_*eff*_(*n* + 1) equal to 0.

Now for small *x*_*eff*_, *f*(*x*_*eff*_) > *x*_*eff*_ and so *x*_*eff*_(*n* + 1) cannot be less than *x*_*eff*_(*n*) for any positive *μ*. For large *x*_*eff*_(*n*), *x*_*eff*_(*n* + 1) ≈ *μCx*_*eff*_(*n*), as *f*(*x*_*eff*_(*n*)) ≈ exp(−*r* *x*_*eff*_(*n*)) which is a very small positive number. So it is again impossible for positive *μ* to yield *x*_*eff*_(*n* + 1) < *x*_*threshold*_. However, if *μ* is negative one can obtain *f*(*x*_*eff*_) + *μCx*_*eff*_ to be less than *x*_*threshold*_ for sufficiently large *x*_*eff*_, as *μCx*_*eff*_ becomes a large negative quantity and dominates the small positive *f*(*x*_*eff*_) term. Fig 10 graphically shows this, and provides an underlying reason for the marked difference in the behaviour of a network with positive and negative *μ*. It is also clear from the map that the population with *μC* = 0 (namely uncoupled populations) will also eventually die out. This is seen in numerical simulations as well (cf. the *C* = 0 flat line in Fig 1).

This argument also holds for Eq 2. If *f*(*x*_*n*_(*i*)) of the *i* ^*th*^ node is very small, then the dynamics is dominated by the interaction term. So *x*_*n*+1_(*i*) ≈ ∑*J*_*ij*_ *x*_*n*_(*j*). If *μ* < 0, the connection matrix is dominated by negative entries, and so the probability of ∑*J*_*ij*_ *x*_*n*_(*j*) being less than zero is high, and consequently the probability of the nodal population mapping to zero is high. This suggests why extinctions are seen to arise for sufficiently negative mean interaction strengths.

To further gauge if the bifurcation diagram obtained numerically in Fig 4(d) can be understood qualitatively using this effective map picture, we study the effective map in Eq 9 under noise. It can be argued that the deviations from the effective dynamics, arising from the spread of *J*_*ij*_ and from the lack of synchronization of *x*_*i*_, can be modelled by random fluctuations about the mean interaction strength *μC* and the value of *x*_*eff*_ in the interaction term. So we study the dynamics given by Eq 9 under the influence of noise, given as:

$$x_{eff}\left(n+1\right)=f\left(x_{eff}\left(n\right)\right)+\left(\mu C+D_{J}\eta \right)\;\left(x_{eff}\left(n\right)+D_{x}\xi \right)$$ (11)

Notice that this noisy effective map captures the renewed growth of a sub-threshold population due to cooperative effects, unlike Eq 9. The bifurcation diagram of the above is shown in Fig 7b. It can be clearly seen that the effective dynamical map, under random noise, qualitatively captures the collective dynamics of the multi-species communities.

### Effect of the degree of variability in inter-species interactions

**Fig 11:**
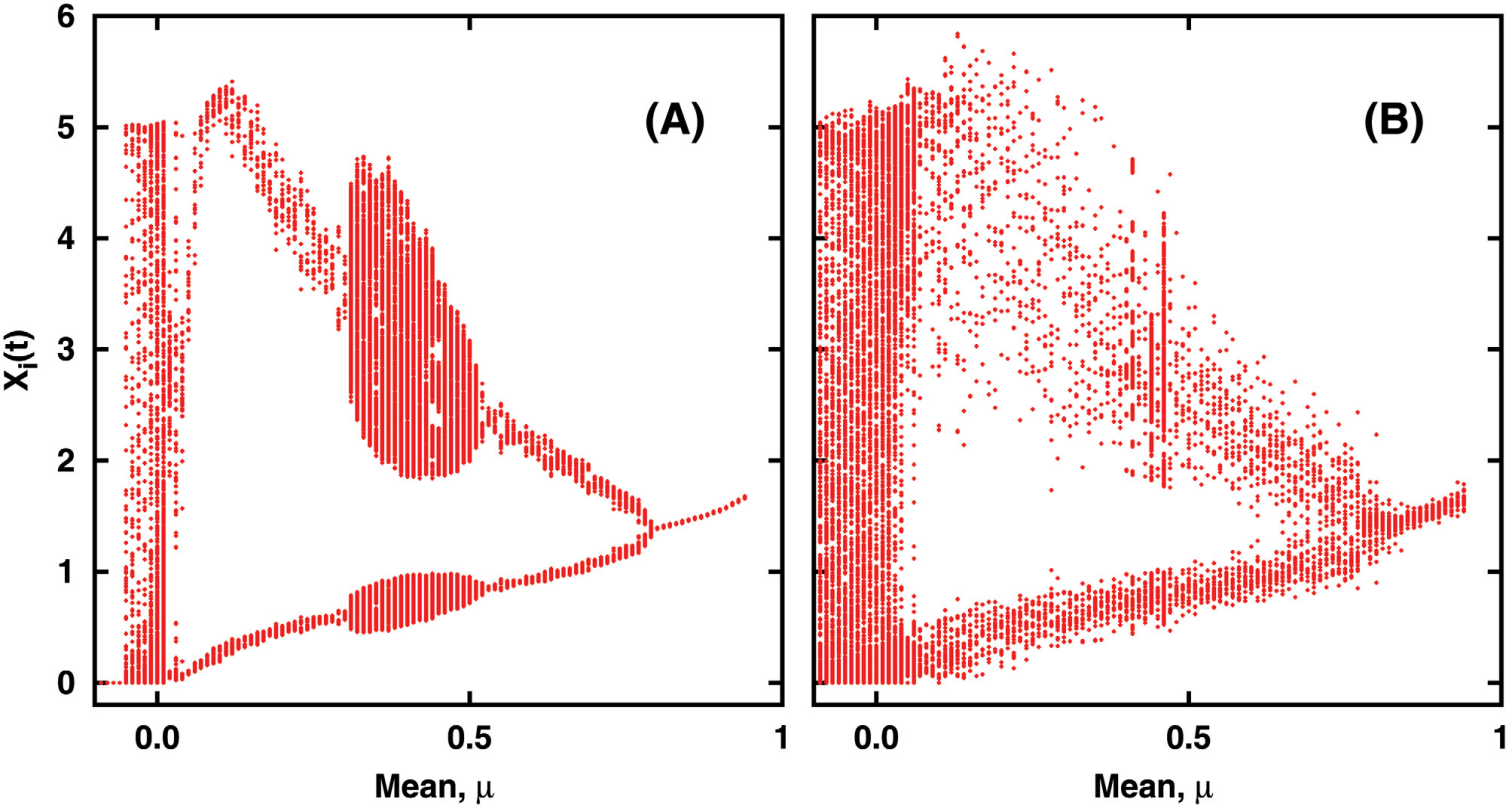
Bifurcation diagram of the population dynamics of all the species as mean interaction strength *μ* is varied, for two different values of standard deviation: (a) *σ* = 0.05 and (b)*σ* = 0.5. For this representative case, the network is taken to be fully connected, i.e. *C* = 1.

Having gained understanding of the collective dynamics of the system in terms of the dynamics of a single effective stochastic map, we now try to understand the effect of the standard deviation *σ* of the connectivity matrix *J* on the dynamics of the multi-species community. It seems reasonable to argue that the strength of the noise term in the effect dynamical map is directly related to *σ*, namely we can associate the stochasticity in the effective map to variability in the inter-species interaction strength across the network. Thus we investigate the changes in the bifurcation structure for two different values of *σ*, which represents different degrees of interaction variability in the network. From Fig 11, one can observe that with increasing *σ*, the bifurcation diagram gets more noisy. This indicates that one can incorporate the variability in interaction structure easily in the effective map.

As discussed earlier, the sign of the interactive term is crucial in determining if a population is driven below threshold or not. So a network is very sensitive to large fluctuations in the distribution of interaction strengths. Namely, large variability around the mean value *μC* in Eq 9 can push the system into the extinction zone, or out of it, when *μ* is close to zero. This accounts for the spread in 〈*N*_*active*_〉 values around *μ* ≈ 0, in the presence of large *σ* in Fig 1.

## Discussions

In summary, we have analyzed the survival properties in ecological networks. In particular, we considered a complex network of populations where the links are given by a random asymmetric connectivity matrix *J*, with fraction 1 − *C* of zero entries, where *C* reflects the over-all connectivity of the system. The non-zero elements are drawn from a Gaussian distribution with mean *μ* and standard deviation *σ*. The signs of the elements *J*_*ij*_ of the connectivity matrix *J* reflect the nature of density-dependent interactions, such as predatory-prey, mutualism or competition, and their magnitude reflect the strength of the interaction. Unlike many earlier studies, we investigate the full range of mean interaction strengths, and do not confine ourselves to the balanced situation which assumes *μ* = 0.

Also note that one can potentially draw a parallel between our model and the system of metapopulations with *density dependent dispersal* [17]. Namely, our system can also be interpreted as a network of metapopulation patches [18], or “a population of populations” [19]. In particular, it can describe a system comprising many spatially discrete sub-populations connected by migrations where inter-patch dispersal is both high enough to ensure demographic connectivity among patches, yet low enough to maintain some degree of independence in local population dynamics [20]. The connectivity matrix in this scenario reflects density dependent dispersal and migration, as is commonly seen in vertebrate and invertebrate populations [21–26].

A problem of vital importance here is understanding how broad features, such as the connectedness and net positive interaction strength, modulates the emergent dynamics in such a network. First, in order to gauge the global stability of the system, we calculate the average number of active nodes, namely number of non-extinct species, in the network. We find that the network transitions from a completely extinct system to one where all nodes are active, as the mean interaction strength goes from negative to positive. This transition, marked distinctly by scaling relations, gets sharper with increasing *C* and decreasing *σ*. This result has much relevance, as realistic ecosystems are unlikely to have a perfect balance of interactions. So understanding the implications of imbalance in interaction types and strengths in the network (namely *μ* ≠ 0) is important.

Another significant observation is that the total population, reflecting the biomass production in a multi-species community, displays distinct non-monotonic scaling behaviour with respect to the product *μC*, implying that survival is dependent not merely on the number of links, but rather on the combination of the sparseness of the connectivity matrix and the net interaction strength. Interestingly, in an intermediate window of positive *μC*, the total population is maximal, indicating that too little or too much positive interactions is detrimental to survival. In fact survival is optimal when the network has intermediate net positive connection strengths. Counter-intuitively then, if positive interactions such as mutualistic or symbiotic links are too dominant, its effect ceases to be beneficial and in fact results in reduction of the total population.

At the local level we observed marked qualitative changes in dynamical patterns, ranging from fixed points to spatiotemporal chaos, under variation of mean *μ* of the interaction strengths. Specifically we found anti-phase clusters of period 2 cycles and the presence of period-2 chaotic bands, in certain windows of mean *μ*. This behaviour is reminiscent of the field experiment conducted by Scheffer et al [16] which showed the existence of self-perpetuating stable states alternating between blue-green algae and green algae. We also studied the correlation between synchronization and survival, and find that synchronization does not necessarily lead to extinction. Lastly, we proposed an effective low dimensional map to capture the behavior of the entire network, and this provides a broad understanding of the interplay of the local dynamical patterns and global stability of the network.
